# Cranial Bone Changes Associated With Intracranial Hypertension in Apert Syndrome: Insights for Early Surgical Intervention

**DOI:** 10.1097/GOX.0000000000006875

**Published:** 2025-06-12

**Authors:** Diego A. Gomez, Isabelle J. Meredith, Skyler K. Palmer, Marius George Linguraru, David Y. Khechoyan, Phuong D. Nguyen, Brooke French, Antonio R. Porras

**Affiliations:** From the * Department of Pediatric Plastic and Reconstructive Surgery, Children’s Hospital of Colorado, Aurora, CO; † Department of Biostatistics and Informatics, Colorado School of Public Health, University of Colorado Anschutz Medical Campus, Aurora, CO; ‡ Sheikh Zayed Institute for Pediatric Surgical Innovation, Children’s National Hospital, Washington, DC; § Departments of Pediatrics, Surgery and Biomedical Informatics, School of Medicine, University of Colorado Anschutz Medical Campus, Aurora, CO.; ¶ Department of Pediatric Neurosurgery, Children’s Hospital Colorado, Aurora, CO.

## Abstract

**Background:**

The presence of intracranial hypertension (ICH) is a key consideration in the surgical management of Apert syndrome (AS). However, cranial signs indicative of ICH are underexplored. We used routinely acquired computed tomographic images to deliver the first quantitative assessment of localized cranial bone and volumetric anomalies associated with ICH in children with AS.

**Methods:**

Children with AS with preoperative computed tomographic scans were retrospectively identified at 2 institutions. Patients with preceding craniofacial intervention were excluded. Local cranial bone thickness, cranial density, and intracranial volume (ICV) anomalies were compared among 3 cohorts: AS, nonsyndromic bicoronal craniosynostosis (NSBC), and normative patients without cranial pathology. Adjustments were made for age and sex.

**Results:**

A total of 671 patients were included (16 AS, 631 normative, and 24 NSBC). All patients with AS displayed bicoronal suture involvement, and 9 showed additional suture fusions. Patients with AS had significantly increased cranial bone thickness and total ICV, as well as significantly decreased cranial bone density and occipital volume compared with the normative cohort. Compared with NSBC, patients with AS demonstrated greater cranial density loss and ICV increase under the frontal and parietal bones, with no significant differences in the occipital region.

**Conclusions:**

Before surgical intervention, children with AS exhibit distinct cranial adaptations to chronic ICH, characterized by increased ICV and decreased cranial bone density, suggesting that earlier surgical intervention may be necessary to prevent the effects of chronic ICH. Furthermore, the predominant volume restriction in the occipital region supports posterior expansion as the initial intervention.

**Takeaways**

**Question:** Do children with Apert syndrome (AS) demonstrate cranial bone changes suggestive of intracranial hypertension before surgical intervention?

**Findings:** At an average of 5 months of age, children with AS demonstrate significantly increased cranial bone thickness, total intracranial volume (ICV), and decreased cranial bone density. Furthermore, these patients demonstrate a significant ICV restriction in the occipital region.

**Meaning:** Routinely acquired computed tomography images can identify signs of chronic intracranial hypertension in children with AS. These patients may benefit from earlier surgical intervention through a posterior vault distraction to correct the significant ICV deficit in the occipital region.

## INTRODUCTION

Apert syndrome (AS) is a rare genetic condition caused by mutations in the fibroblast growth factor receptor 2 and characterized by craniosynostosis, midface hypoplasia, and complex syndactyly.^1,2^ Hallmark features include a long and narrow head, midface retrusion, and proptosis.^1^ Multisuture craniosynostosis is nearly universal, with bilateral coronal involvement affecting most patients and frequent sagittal and/or lambdoid involvement.^3^

Management of AS is complex and requires a multidisciplinary approach. At birth, close attention to cerebral, respiratory, and ocular emergencies is needed, with intracranial hypertension (ICH) being a critical challenge reported in 45%–83% of patients.^4,5^ The etiology of ICH is multifactorial and results from skull growth restriction, hydrocephalus, and airway obstruction causing craniocerebral disproportion.^6^ Without intervention, ICH can cause or exacerbate neurodevelopmental delays and cognitive impairment, highlighting the importance of early diagnosis. Although some institutions perform anterior or posterior decompression within the first year of life,^7,8^ others delay surgical treatment until ICH is diagnosed,^4,9^ and the optimal time of intervention remains a matter of debate.

Clinical signs of acute ICH such as somnolence, irritability, vomiting, headaches, or cranial nerve palsy warrant urgent surgical treatment to prevent neurological complications.^7,8,10^ However, most patients with AS present chronic or nonacute ICH with subtle and nonspecific presentation, and its identification is challenging.^4,11^ Clinical signs of ICH, such as papilledema, are unreliable in children younger than 8 years^12^ and are present in only up to 51% of patients with AS.^13^ Invasive monitoring techniques such as transcranial intracranial pressure monitoring and lumbar punctures remain the most accurate method for detecting ICH. However, they are avoided because of the lack of widely accepted thresholds in pediatric populations^9^ and significant risks, including mechanical malfunction, cerebrospinal fluid leak, intracranial hemorrhage, and infection.^14^

Recent studies demonstrate that routinely acquired computed tomographic (CT) images can systematically identify cranial bone changes associated with ICH in pediatric populations. Specifically, chronic ICH has been correlated with decreased calvarial bone density and increased skull thickness in pediatric patients with both syndromic and nonsyndromic forms of craniosynostosis,^15^ and in those with chronic ICH unrelated to craniosynostosis.^16^ These cranial anomalies differ from the observations of bone thinning in acute ICH in adults^17^ and may reflect differential cranial remodeling capabilities in children.^16,18,19^

Although previous research supports the early identification of ICH in patients with different forms of craniosynostosis, no quantitative studies examine the relationship of ICH with local volumetric cranial growth constraints and the resulting compensatory local overgrowth, which could inform surgical management. Moreover, no studies explore the distinctive phenotype of AS compared with nonsyndromic craniosynostosis, which may reduce institutional management variability. Posterior vault distraction osteogenesis (PVDO) and fronto-orbital advancement (FOA) remain common initial approaches, although the optimal strategy remains a matter of debate.^7^

The goal of this study was to provide a comprehensive and quantitative characterization of the unique calvarial anomalies in children with AS. Specifically, we sought to quantify local bone density and thickness anomalies as indicators of chronic ICH,^15,16^ the abnormal distribution of local intracranial volume anomalies that are essential for surgical planning, and their relationships to provide a quantitative basis to inform surgical management strategies for this population.

## METHODS

### Data

We performed a retrospective review of patients with a clinically or genetically confirmed diagnosis of AS at Children’s Hospital of Colorado (protocol no. 20-1563) and Children’s National Hospital (protocol no. 3792). Non-surgically treated children younger than 18 years of age with available preoperative CT scans were included. Patients with preceding craniofacial surgery, shunts, or craniofacial pathology unrelated to AS such as trauma or cranial tumors were excluded. Images with an in-plane resolution larger than 0.5 mm or slice thickness larger than 1.5 mm were excluded to reduce image variability and avoid bias in local bone quantification.^16^

To investigate the specific phenotype of craniosynostosis associated with AS, we used 2 other datasets as references. First, we identified 631 patients aged 0–3 years, matching the age range in the AS cohort, from a cross-sectional dataset of 1018 subjects without cranial pathology as a normative reference.^16^ These were patients referred to the emergency room after trauma and for whom clinical and radiological evaluation discarded any cranial anomalies. Separately, we retrospectively identified patients with nonsyndromic bicoronal craniosynostosis (NSBC) using the same inclusion and exclusion criteria as for our patients with AS. This patient group was used as a nonsyndromic reference of patients with a fusion of the same cranial sutures as our patients with AS to study the specific phenotype of AS.

### Image Processing

We used existing public methods to automatically segment the calvaria and label the 5 major cranial bones from each CT image: left and right frontal, left and right parietal, and occipital.^20,21^ Any inaccuracies in cranial bone segmentation were manually corrected using ITK-SNAP. After standardizing the anatomical representation of the cranium and establishing anatomical correspondences using prior spherical mapping methods,^16,20^ local bone density in Hounsfield units and thickness in millimeters were calculated at each point of the calvaria. Local bone density was calculated as the average density between the inner and outer cranial bone surfaces.^15,16^ Before bone density calculations, images were first resampled to a uniform in-plane resolution of 0.5 mm and slice thickness of 1.5 mm using linear interpolation to reduce data variability caused by partial volume effects.^16^

### Statistical Analysis

Similar to previous work,^22^ we used our normative statistical model of cranial bone development to calculate cranial anomalies as local distances between the segmented cranial surface from the CT image of each patient and their age- and sex-specific personalized normative statistical reference in millimeters.^23^ Then, we spatially integrated those distances to quantify the volumetric anomalies as deficit or excess of volume under each cranial bone in milliliters. Similarly, we calculated cranial thickness and density anomalies as the local differences between the thickness and density calculated from the CT image of each patient and their age- and sex-specific personalized normative statistical reference in millimeters and Hounsfield units, respectively.^22,23^ All values were averaged both within the entire calvaria and within each cranial bone. Statistical differences were then evaluated between patients with AS, patients with NSBC, and normative subjects, using pairwise nonparametric Mann-Whitney *U* tests.

## RESULTS

### Patient Data

A total of 16 patients with AS (8 men, 8 women, age 4.84 ± 8.37 mo, age range 0–3 y) met our inclusion criteria. We identified 631 subjects 0–3 years of age from the normative cohort of 1018 patients (331 men, 300 women, age 11.81 ± 9.09 mo) and 24 patients with NSBC (7 men, 17 women, age 6.34 ± 7.42 mo) within the same age range (Table 1).

**Table 1.** Patient Demographics

	AS	NSBC	Normative
Age, mo	4.84 ± 8.37	6.34 ± 7.42	11.81 ± 9.09
No. patients	16	24	631
Male, n (%)	8 (50)	7 (29)	331 (52)
Female, n (%)	8 (50)	17 (71)	300 (48)
Fused sutures, n (%)			
Bicoronal	16 (100)	24 (100)	—
Sagittal	1 (6)	4 (17)	—
Metopic	2 (13)	2 (8)	—
Right lambdoid	7 (44)	1 (4)	—
Left lambdoid	2 (13)	2 (8)	—
Squamosal	4 (25)	1 (4)	—

### Cranial Anomalies in Patients With AS

**Fig. 1.**
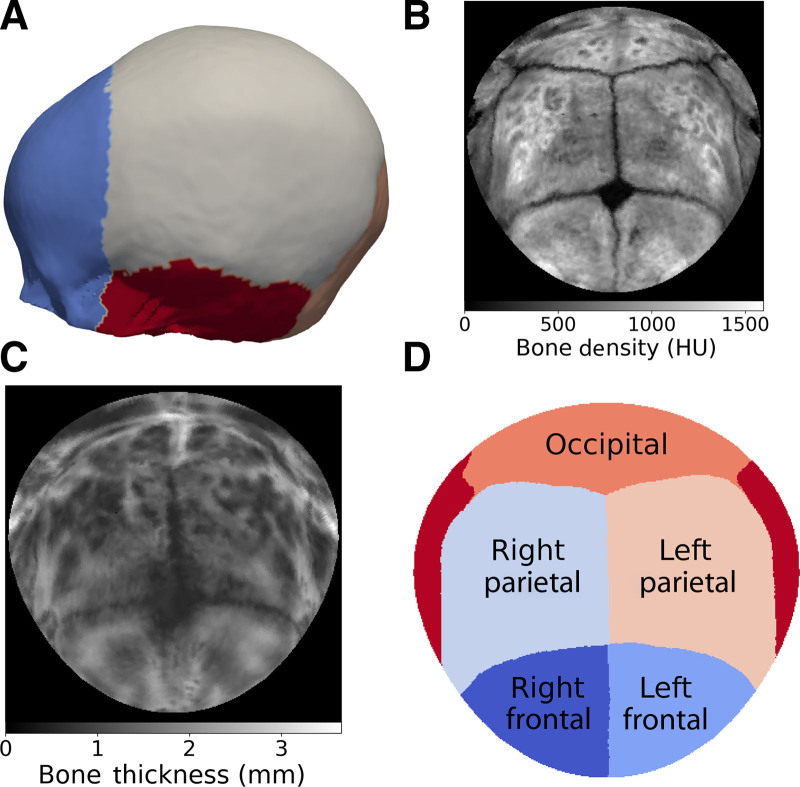
Standardized representation of the calvaria. A, Cranial surface segmented from a CT image with color-coded bone labels. Standardized 2-dimensional representation using spherical mapping of the average bone density in Hounsfield units (HU) (B) and thickness in millimeters (C) for a 1.5-year-old normative subject. D, Location of every major cranial bone.

**Fig. 2.**
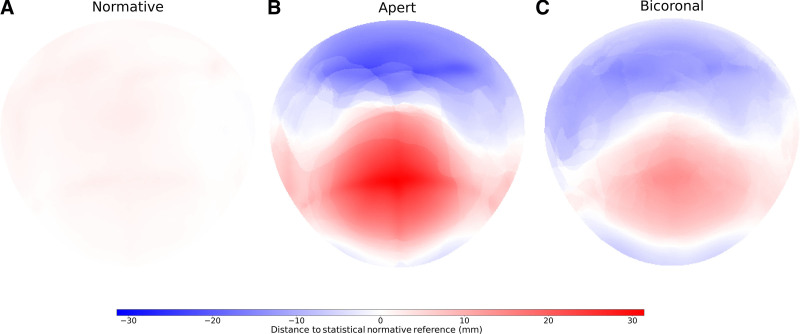
Local volumetric anomalies. Average local volumetric anomalies represented as signed distances to a personalized statistical normative reference in millimeters for the normative subjects (A), patients with AS (B), and patients with NSBC (C). Negative values represent volume loss, and positive values indicate volume gain.

**Fig. 3.**
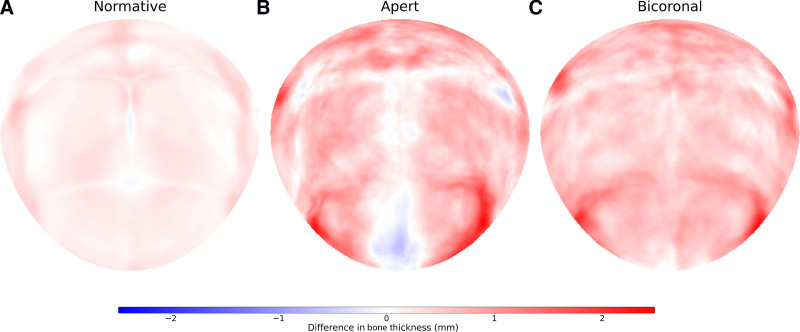
Local bone thickness anomalies. Average local bone thickness anomalies in millimeters for the normative subjects (A), patients with AS (B), and patients with NSBC (C). Negative values indicate bone thinning, and positive values indicate bone thickening.

**Fig. 4.**
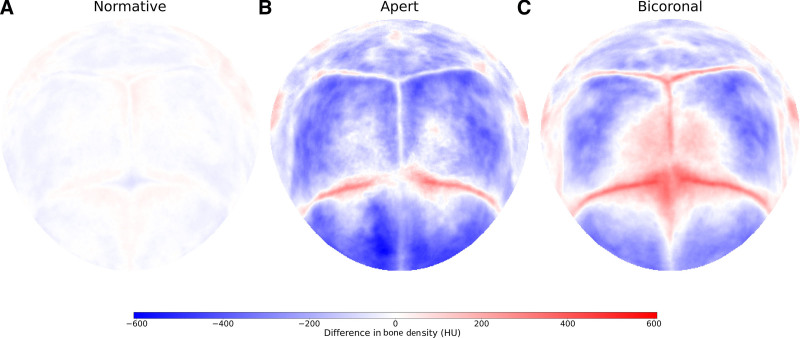
Local bone density anomalies. Average local cranial bone density anomalies in Hounsfield units (HU) for the normative subjects (A), patients with AS (B), and patients with NSBC (C). Negative values indicate lower density than normative, and positive values indicate higher density than normative.

Figure 1 demonstrates the standard representation of the calvaria. Figures 2, 3, and 4 illustrate the local cranial volumetric, bone thickness, and density anomalies for our patients with AS and NSBC, and our normative subjects. Table 2 presents the average age- and sex-matched cranial differences compared with normative data for patients with AS and patients with NSBC.

**Table 2.** Cranial Anomalies Quantified in Patients with AS and NSBC

	AS	NSBC	*P*
Volumetric anomalies, mL			
Calvaria	147.23 ± 130.69*	0.07 ± 80.83	<0.001
Left frontal bone	41.17 ± 28.54*	14.44 ± 13.95*	0.002
Right frontal bone	33.99 ± 18.76*	10.00 ± 12.96†	<0.001
Left parietal bone	92.69 ± 87.30*	23.55 ± 30.76†	0.005
Right parietal bone	91.74 ± 66.09*	17.43 ± 36.66	<0.001
Occipital bone	−48.73 ± 27.15*	−41.99 ± 33.97*	0.33
Thickness anomalies, mm			
Calvaria	0.53 ± 0.21*	0.57 ± 0.31*	0.67
Left frontal bone	0.74 ± 0.37*	0.70 ± 0.43*	0.67
Right frontal bone	0.57 ± 0.39*	0.61 ± 0.47*	0.75
Left parietal bone	0.52 ± 0.28*	0.47 ± 0.32*	0.65
Right parietal bone	0.51 ± 0.20*	0.46 ± 0.30*	0.71
Occipital bone	0.67 ± 0.31*	0.66 ± 0.47*	0.86
Density anomalies (Hounsfield units)			
Calvaria	−158.43 ± 76.40*	−57.34 ± 126.43*	0.011
Left frontal bone	−247.51 ± 77.76*	−116.32 ± 159.96*	0.001
Right frontal bone	−267.13 ± 91.32*	−122.45 ± 155.04*	<0.001
Left parietal bone	−187.47 ± 109.22*	−71.86 ± 145.12†	0.015
Right parietal bone	−184.29 ± 125.33†	−54.92 ± 151.22†	0.015
Occipital bone	−96.57 ± 120.04*	−59.30 ± 159.80	0.42

*P* values are calculated between patients with AS and NSBC using a Mann-Whitney *U* test.

* Significantly different anomalies compared with the group of normative subjects for *P* < 0.01.

† Significantly different anomalies compared with the group of normative subjects for *P* < 0.05.

We found a significantly increased intracranial volume (ICV) in patients with AS compared with normative subjects (147.23 ± 130.59 mL; *P* < 0.01). Locally, a significant ICV decrease was observed under the occipital bone of patients with AS (−48.73 ± 27.15 mL; *P* < 0.01), as opposed to the significant volume increases observed under the frontal and parietal bones (Fig. 2B).

We found a significant increase in bone thickness in patients with AS compared with normative subjects, both globally and locally at each cranial bone. Global and localized cranial density was found to be significantly lower in patients with AS compared with normative subjects. This density decrease showed a posterior-to-anterior severity pattern, with higher density loss observed in the parietal bones compared with the occipital bone, and in the frontal bones compared with the parietal bones. Figures 3B and 4B show more pronounced cranial thickening at the fused coronal sutures, and a generalized density decrease at all locations except in the fused sutures. Figure 3B also indicates local bone thinning around the metopic suture in AS.

### Cranial Anomalies in Patients With NSBC

Patients with NSBC did not show significantly different ICV from normative subjects (0.07 ± 80.83 mL; *P* > 0.05), although significant differences in local volumetric distributions were identified with significant frontal volume overdevelopment and occipital volume underdevelopment (Table 2 and Fig. 2C). Patients with NSBC showed significantly increased calvarial thickness and decreased cranial bone density relative to normative subjects both globally and locally. Figures 3C and 4C depict cranial thickening in these patients around the fused coronal sutures, and an overall density decrease at all cranial locations except around the fused sutures and neighboring regions of the anterior fontanelle.

### Differences Between AS and NSBC

Compared with patients with NSBC, patients with AS exhibited a significantly increased ICV (*P* < 0.001) driven by significant volume gains under the frontal and parietal bones. No volumetric differences were identified under the occipital bone (Table 2). Figures 2B and C show similar but more severe volume anomalies in AS compared with NSBC.

Cranial thickness was not statistically different between patients with AS and NSBC either globally or locally at any cranial bone. Although patterns of abnormal thickening were similar, metopic thinning was only seen in AS (Figs. 3B, C).

Finally, an overall cranial bone density decrease was observed in patients with AS compared with patients with NSBC. This density decrease was significant at the frontal and parietal bones, but not at the occipital bone. Moreover, although similar posterior patterns of local density anomalies were observed between these groups with different levels of severity, patients with NSBC seemed to present increased bone density surrounding the anterior fontanelle that was not observed in patients with AS (Figs. 4B, C).

## DISCUSSION

We performed the first comprehensive and quantitative study of the local cranial volume, thickness, and density anomalies and their relationships in patients with AS that had only been clinically and/or qualitatively described previously.^24^ Research on craniofacial manifestations in AS has largely centered on broad intracranial features such as the cranial fossa, midface, and orbits, with no analysis of localized cranial vault morphology to date.^25–27^ Previous studies have demonstrated that ICV in AS is normal at birth, and then progresses to 3 SDs above normal by 4 months—a trend that occurs irrespective of cranial vault surgery.^28^ Whereas ICV increases, head circumference percentile decreases with age.^24^ Despite their larger ICV, children with AS remain at high risk of ICH due to early suture fusion and hydrocephalus, resulting in craniocerebral disproportion and subsequent elevations in intracranial pressure in up to 83% of patients.^4^ As a result, these patients still benefit from cranial vault expansion to mitigate the risk of intracranial pressure elevation.

Despite phenotypic similarities between patients with AS and patients with similar nonsyndromic forms of craniosynostosis, our results quantitatively show a distinct phenotype of AS. The cranial phenotype of AS is traditionally described as a severe form of bicoronal craniosynostosis with craniocerebral disproportion.^25^ Indeed, our results quantitatively show that patients with AS had significantly increased ICV compared with the normative population unlike patients with NSBC. When examining local volume anomalies, we quantified similar posterior volumetric deficits under the occipital bone between patients with AS and patients with NSBC, suggesting the increased ICV in AS is due to more severe anterior volumetric overdevelopment. Volume overdevelopment typically occurs parallel to the fused sutures and is driven by the increased pressure from constrained brain growth. Studies using invasive monitoring techniques have demonstrated a high rate of ICH in patients with AS before cranial vault surgery, ranging from 50%^11^ to 83%.^4^ Hence, although our institution does not use invasive monitoring due to the associated risks, and these measurements were not available in this retrospective study, our approach found similar rates of ICH to prior studies.^4,11^ Separately, a recent study has shown that cranial bone thickening and density loss are indicators of chronic ICH.^16^ Our findings align with prior studies and provide additional evidence of ICH in untreated patients with AS.

Given the previously reported link between chronic pediatric ICH and decreased cranial bone density and increased cranial thickening,^15,16^ we studied the local distributions of these cranial signs of ICH and their relationship with the abnormal volumetric distributions in patients with AS and patients with NSBC. We identified significant cranial bone thickening in both patient groups, suggesting an adaptive response to early suture fusion. Importantly, differences in cranial thickening between patients with AS and NSBC were not significant, suggesting that they may not be related to the genetic mutations causing AS.

Both children with AS and children with NSBC displayed decreased cranial density throughout the calvaria compared with the normative population, most pronounced in the frontal bones and significantly more severe in patients with AS. This combination of calvarial thickening and decreased cranial bone density has been demonstrated to be associated with chronic ICH of diverse etiology.^15,16^ These findings support prior studies showing a higher prevalence of chronic ICH in patients with coronal suture involvement compared with midline craniosynostosis, and the higher risk of ICH in patients with AS before treatment.^15^ Patients with AS distinctively exhibited metopic suture thinning, which may represent a response to greater intracranial pressure causing frontal bone separation (Fig. 3B).

The average age of first cranial expansion in AS is around 7.7 months, ranging from 1 month to 4 years and 5 months.^4,29^ This is influenced by the presence of ICH, cranial and midfacial anomalies, and other associated conditions such as Chiari malformations.^29^ Additionally, different institutions have varying protocols for the timing of cranial surgery. In our study, the average age of image acquisition in the AS cohort was 4.84 months (range 0–3 y), suggesting that before 5 months of age, children with AS display signs of chronic ICH and may, therefore, benefit from earlier surgical intervention to prevent neurological sequelae.

The posterior restriction identified in our study may explain why PVDO is increasingly becoming the first-line intervention for most syndromic patients.^7,30,31^ Recent studies have demonstrated that PVDO yields greater increases in ICV compared with fronto-orbital approaches, effectively reducing the incidence of tonsillar herniation and papilledema while offering a similar safety profile.^32^ Furthermore, PVDO may reduce future surgical burdens, with 1 study on 13 patients with AS demonstrating that children with initial PVDO had a reduced need for subsequent FOA.^33^ Similarly, studies have also reported higher reoperation rates following primary FOA compared with PVDO in patients with AS, suggesting limitations in addressing posterior growth restrictions.^34,35^

This study was limited by the inherent constraints of CT imaging resolution and the impact of partial volume effects on quantitative analysis. Although we sought to mitigate these limitations by resampling all images to a uniform resolution and implementing strict inclusion and exclusion criteria, the constraints of voxel size persist. Each voxel could capture multiple tissue types—thus compromising the precision of tissue density and thickness measurements. This averaging effect may reduce the sensitivity of our cranial density and calvarial thickness assessments, which may underestimate the true extent of morphological variation in AS. Moreover, our statistical analysis did not capture changes in the skull base due to imaging protocols that focused primarily on the cranial vault. The skull base is known to undergo distinct morphological alterations in syndromic craniosynostosis, contributing to functional complications and airway obstruction. Although prior studies have documented skull base abnormalities in AS, our inability to include this region in our analysis limits the comprehensiveness of our craniofacial phenotype characterization.^25^ Future work should prioritize the inclusion of the skull base, as well as leverage 3-dimensional volumetric and morphometric analyses of the calvaria in combination with the skull base.

Another limitation relates to the retrospective nature of this study. As invasive intracranial pressure measurements are not standard of care for patients with AS at our institution, we were unable to confirm the presence or absence of ICH in this cohort. However, the established high prevalence of ICH in this population, combined with the presence of known cranial bone changes associated with ICH, supports our hypothesis that these cranial bone changes are representative of underlying ICH. Future studies incorporating radiological and clinical data would strengthen this association. Finally, although our cohort of patients with NSBC lacked an identified genetic etiology, it remains plausible that an undetected or unrecognized genetic basis contributed to the condition in some individuals, potentially explaining the increased bone density along the anterior fontanelle in this cohort.

Our findings have potential clinical implications for diagnosing and managing ICH in AS. Given the challenges of noninvasively assessing intracranial pressure in AS, our study suggests that analysis of routinely acquired CT imaging—specifically, quantification of calvarial thickness, cranial density, and regional ICV—may serve as valuable adjunctive tools for identifying early intracranial pressure elevation. Furthermore, the presence of cranial bone changes associated with ICH at an average age of 4.84 months suggests that these patients display signs of ICH before surgery, potentially benefiting from earlier surgical intervention compared with the average intervention at 7 months of age.^29^ Our findings support the growing body of literature recommending performing posterior cranial vault distraction earlier, between 3 and 6 months, to proactively address ICH before it develops.^31,36^ Finally, the presence of a significant ICV deficit in the occipital region supports the use of posterior vault distraction as an initial intervention to prevent ICH. Future research could assess how early cranial vault characteristics predict long-term neurodevelopmental outcomes.

## CONCLUSIONS

Children with AS exhibit distinctive cranial bone adaptations before surgery, including increased ICV, calvarial thickening, and decreased density driven by genetic mutations and craniosynostosis-related growth constraints. Our findings suggest that these patients display signs of chronic ICH by 5 months of age and may potentially benefit from earlier cranial vault surgery to prevent neurological complications. Moreover, the predominant ICV deficit in the occipital region supports the use of posterior vault distraction as a first surgical intervention in this population. The use of CT imaging offers a noninvasive means to identify structural changes associated with ICH, enhancing clinical decision-making, optimizing surgical timing, and potentially improving outcomes for pediatric patients with AS.

## DISCLOSURES

The authors have no financial interest to declare in relation to the content of this article. Research reported in this publication was supported by the National Institute of Dental & Craniofacial Research of the National Institutes of Health under Award Numbers R21DE031824 and R00DE027993, and by National Institutes of Health/National Center for Advancing Translational Sciences Colorado Clinical and Translational Science Award under grant number UM1 TR004933. The content is solely the responsibility of the authors and does not necessarily represent the official views of the National Institutes of Health.

## References

[1] Alam MK Alfawzan AA Srivastava KC. Craniofacial morphology in Apert syndrome: a systematic review and meta-analysis. Sci Rep. 2022;12:5708. 35383244 10.1038/s41598-022-09764-y PMC8983770

[2] Wilkie AO Slaney SF Oldridge M. Apert syndrome results from localized mutations of FGFR2 and is allelic with Crouzon syndrome. Nat Genet. 1995;9:165–172. 7719344 10.1038/ng0295-165

[3] Bannink N Joosten KF van Veelen ML. Papilledema in patients with Apert, Crouzon, and Pfeiffer syndrome: prevalence, efficacy of treatment, and risk factors. J Craniofac Surg. 2008;19:121–127. 18216676 10.1097/SCS.0b013e31815f4015

[4] Marucci DD Dunaway DJ Jones BM. Raised intracranial pressure in Apert syndrome. Plast Reconstr Surg. 2008;122:1162–1168. 18827651 10.1097/PRS.0b013e31818458f0

[5] Renier D Lajeunie E Arnaud E. Management of craniosynostoses. Childs Nerv Syst. 2000;16:645–658. 11151714 10.1007/s003810000320

[6] Anderson PJ Netherway DJ Abbott AH. Analysis of intracranial volume in Apert syndrome genotypes. Pediatr Neurosurg. 2004;40:161–164. 15608488 10.1159/000081933

[7] Raposo-Amaral CE Denadai R Oliveira YM. Apert syndrome management: changing treatment algorithm. J Craniofac Surg. 2020;31:648–652. 31895846 10.1097/SCS.0000000000006105

[8] Warren SM Proctor MR Bartlett SP. Parameters of care for craniosynostosis: craniofacial and neurologic surgery perspectives. Plast Reconstr Surg. 2012;129:731–737. 22373978 10.1097/PRS.0b013e3182412a50

[9] Breik O Mahindu A Moore MH. Apert syndrome: surgical outcomes and perspectives. J Craniomaxillofac Surg. 2016;44:1238–1245. 27378001 10.1016/j.jcms.2016.06.001

[10] Mathijssen IM. Guideline for care of patients with the diagnoses of craniosynostosis: working group on craniosynostosis. J Craniofac Surg. 2015;26:1735–1807. 26355968 10.1097/SCS.0000000000002016 PMC4568904

[11] Spruijt B Joosten KFM Driessen C. Algorithm for the management of intracranial hypertension in children with syndromic craniosynostosis. Plast Reconstr Surg. 2015;136:331–340. 25909300 10.1097/PRS.0000000000001434

[12] Tuite GF Chong WK Evanson J. The effectiveness of papilledema as an indicator of raised intracranial pressure in children with craniosynostosis. Neurosurgery. 1996;38:272–278. 8869054 10.1097/00006123-199602000-00009

[13] Kim SY Choi JW Shin HJ. Reliable manifestations of increased intracranial pressure in patients with syndromic craniosynostosis. J Craniomaxillofac Surg. 2019;47:158–164. 30497950 10.1016/j.jcms.2018.10.021

[14] Tamburrini G Caldarelli M Massimi L. Intracranial pressure monitoring in children with single suture and complex craniosynostosis: a review. Childs Nerv Syst. 2005;21:913–921. 15871027 10.1007/s00381-004-1117-x

[15] Chaij J Liu J French B. Investigation of cranial bone changes indicative of increased intracranial pressure in diverse phenotypes of craniosynostosis. Plast Reconstr Surg Glob Open. 2025;13:e6618. 40115040 10.1097/GOX.0000000000006618 PMC11925430

[16] Liu J Chaij J Linguraru MG. Cranial bone thickness and density anomalies quantified from CT images can identify chronic increased intracranial pressure. Neuroradiology. 2024;66:1817–1828. 38871879 10.1007/s00234-024-03393-0 PMC11424726

[17] Barke M Castro HM Adesina OO. Thinning of the skull base and calvarial thickness in patients with idiopathic intracranial hypertension. J Neuroophthalmol. 2022;42:192–198. 35195543 10.1097/WNO.0000000000001504

[18] Rabbani CC Patel JM Nag A. Association of intracranial hypertension with calvarial and skull base thinning. Otol Neurotol. 2019;40:e619–e626. 31045889 10.1097/MAO.0000000000002249

[19] Fong KD Warren SM Loboa EG. Mechanical strain affects dura mater biological processes: implications for immature calvarial healing. Plast Reconstr Surg. 2003;112:1312–1327. 14504515 10.1097/01.PRS.0000079860.14734.D6

[20] PorrasPerez AR Keating R Lee J. Predictive statistical model of early cranial development. IEEE Trans Biomed Eng. 2022;69:537–546. 34324420 10.1109/TBME.2021.3100745 PMC8776594

[21] Liu J Xing F Shaikh A. Joint cranial bone labeling and landmark detection in pediatric CT images using context encoding. IEEE Trans Med Imaging. 2023;42:3117–3126. 37216247 10.1109/TMI.2023.3278493 PMC10760565

[22] Porras AR Tu L Tsering D. Quantification of head shape from three-dimensional photography for presurgical and postsurgical evaluation of craniosynostosis. Plast Reconstr Surg. 2019;144:1051e–1060e. 10.1097/PRS.0000000000006260 PMC6905129 31764657

[23] Liu J Elkhill C LeBeau S. Data-driven normative reference of pediatric cranial bone development. Plast Reconstr Surg Glob Open. 2022;10:e4457. 35983543 10.1097/GOX.0000000000004457 PMC9377678

[24] Breakey RWF Knoops PGM Borghi A. Intracranial volume and head circumference in children with unoperated syndromic craniosynostosis. Plast Reconstr Surg. 2018;142:708e–717e. 10.1097/PRS.0000000000004843 30113443

[25] Lu X Forte AJ Wilson A. Cranial fossa volume and morphology development in apert syndrome. Plast Reconstr Surg. 2020;145:790e–802e. 10.1097/PRS.0000000000006679 32221226

[26] Lu X Sawh-Martinez R Jorge Forte A. Classification of subtypes of Apert syndrome, based on the type of vault suture synostosis. Plast Reconstr Surg Glob Open. 2019;7:e2158. 31044122 10.1097/GOX.0000000000002158 PMC6467634

[27] Forte AJ Alonso N Persing JA. Analysis of midface retrusion in Crouzon and Apert syndromes. Plast Reconstr Surg. 2014;134:285–293. 25068327 10.1097/PRS.0000000000000360

[28] Gosain AK McCarthy JG Glatt P. A study of intracranial volume in Apert syndrome. Plast Reconstr Surg. 1995;95:284–295. 7824608 10.1097/00006534-199502000-00008

[29] Fearon JA Ditthakasem K Harrison L. Thirty-year experience treating syndromic craniosynostosis: long-term outcomes following cranial expansions. Plast Reconstr Surg. 2025;155:131–137. 38589995 10.1097/PRS.0000000000011460

[30] Al-Shaqsi S Ching JA Novak CB. Morphometric analysis and outcomes following posterior cranial vault distraction in syndromic and multisuture craniosynostosis. J Plast Reconstr Aesthet Surg. 2023;87:379–386. 37935093 10.1016/j.bjps.2023.10.101

[31] Humphries LS Swanson JW Bartlett SP. Craniosynostosis: posterior cranial vault remodeling. Clin Plast Surg. 2021;48:455–471. 34051898 10.1016/j.cps.2021.03.001

[32] Spruijt B Rijken BFM den Ottelander BK. First vault expansion in Apert and Crouzon-Pfeiffer syndromes: front or back? Plast Reconstr Surg. 2016;137:112e–121e. 10.1097/PRS.0000000000001894 26368328

[33] Wu M Barnett SL Massenburg BB. Early posterior vault distraction osteogenesis changes the syndromic craniosynostosis treatment paradigm: long-term outcomes of a 23-year cohort study. Childs Nerv Syst. 2024;40:2811–2823. 38904767 10.1007/s00381-024-06465-x PMC11322207

[34] Wall SA Goldin JH Hockley AD. Fronto-orbital re-operation in craniosynostosis. Br J Plast Surg. 1994;47:180–184. 8193856 10.1016/0007-1226(94)90051-5

[35] Wong GB Kakulis EG Mulliken JB. Analysis of fronto-orbital advancement for Apert, Crouzon, Pfeiffer, and Saethre-Chotzen syndromes. Plast Reconstr Surg. 2000;105:2314–2323. 10845283 10.1097/00006534-200006000-00002

[36] Taylor JA Bartlett SP. What’s new in syndromic craniosynostosis surgery? Plast Reconstr Surg. 2017;140:82e–93e. 10.1097/PRS.0000000000003524 28654610

